# Exploring the meaning of life among Chinese adolescents with nephrotic syndrome: determinants and psychological correlates

**DOI:** 10.3389/fpsyg.2024.1384374

**Published:** 2024-07-12

**Authors:** Ying Liang, Ruijie Huang, Xiuzhuang Luo, Shuyan Mo, Zhichuan He, Junhong Tian, Lijuan Yang, Yi Xue, Xiaomi Luo

**Affiliations:** ^1^Youjiang Medical University for Nationalities, Baise, China; ^2^The Affiliated Hospital of Youjiang Medical University for Nationalities, Baise, China; ^3^Qian Xi Nan People's Hospital, Xingyi, China

**Keywords:** adolescent, nephrotic syndrome, meaning of life, subjective wellbeing, psychological security, chronic disease

## Abstract

**Background:**

Adolescents with Nephrotic Syndrome (NS) confront unique challenges that influence their Meaning of Life (MOL), a concept crucial for psychological resilience. The chronic nature of NS and its associated burdens necessitate a deeper exploration of MOL and its determinants within this demographic, previously underexamined in research. This study aims to investigate MOL among Chinese adolescents with NS, identifying key factors influencing their sense of meaning and examining the interrelations with Subjective Well-Being (SWB) and Psychological Security (PS).

**Methods:**

Employing a cross-sectional survey design, we analyzed 150 adolescents with NS from Baise City, Guangxi, using the Purpose in Life (PIL) scale alongside PS and SWB scales. Sociodemographic and disease-related variables were assessed for their impact on MOL. Data analysis included descriptive statistics, multiple linear regression, and correlation analyses to explore predictors of MOL and its association with SWB and PS.

**Results:**

A significant proportion (62.0%) of participants exhibited MOL scores below the threshold, indicating diminished life meaning. Critical factors impacting MOL included ‘left-behind’ status, family structure, educational disruptions, and NS duration. Strong correlations emerged between MOL, SWB (*r* = 0.70, *p* < 0.01), and PS, highlighting the interdependence of these psychological dimensions. The study further revealed ‘Proactivity’ as a vital component of MOL, suggesting that despite their challenges, adolescents with NS maintain a keen engagement with life. Key aspects such as ‘Certainty in Control’, ‘Mood of Melancholy or Pleasure’, and ‘Vitality’ emerged as crucial for intervention.

**Conclusion:**

The findings underline the profound impact of NS on adolescents’ MOL, influenced by both sociodemographic and disease-specific factors. By identifying key areas for psychological intervention, this study contributes to the holistic care and treatment of adolescents with NS, advocating for integrated approaches that address their unique challenges and support systems.

## 1 Introduction

The exploration of the Meaning of Life (MOL) in adolescents grappling with Nephrotic Syndrome (NS) offers a unique perspective in understanding this chronic condition. Viktor Frankl’s concept of MOL Frankl (1985) emphasizes the intrinsic value of individual existence and self-worth, highlighting that even in extreme suffering and adversity, individuals can find significance in their experiences, serving as a source of strength and motivation (Rosenfeld et al., 2018; Parker, 2022). This is particularly relevant for adolescents with NS, where the chronic nature of the disease, its physical health impacts, and the psychological stress it imposes create a set of unique challenges. Adolescents, as they navigate the transitional phase from childhood to adulthood, are particularly sensitive to issues of identity, self-worth, and future aspirations. The chronic illness experience can disrupt normal development, leading to heightened existential questions and a search for meaning. Unlike adults, adolescents are still forming their sense of self and place in the world, which can make the quest for MOL more acute and complex.

NS, characterized by increased glomerular basement membrane permeability, leads to proteinuria, hypoproteinemia, hypercholesterolemia, and edema. It affects 2 to 7 per 100,000 children and adolescents annually with a prevalence of about 16 per 100,000 (Eddy and Symons, 2003; Pasini et al., 2017), and can progress to severe complications such as infections, electrolyte imbalances, and potentially end-stage renal disease (ESRD) (Filler et al., 2003; Pasini et al., 2017; Politano et al., 2020). In the context of NS, adolescents might struggle with feelings of isolation due to frequent hospital visits and physical limitations, which can affect their social interactions and self-image. The MOL for these young individuals often involves finding a balance between managing their illness and pursuing their personal goals and dreams. Understanding and supporting the MOL in adolescents with NS can thus play a critical role in their overall well-being and resilience, helping them to maintain a positive outlook despite their health challenges.

The impact of NS on adolescents extends beyond physical symptoms to psychological burdens. The uncertainty and chronicity of NS, coupled with the side effects of intensive medication regimes, often lead to heightened levels of anxiety, pain, and depression, exacerbating psychological distress. This is especially pronounced in adolescents, who are more susceptible to severe negative psychological and behavioral issues compared to their healthy peers (Guo et al., 2021). In this context, MOL becomes critically important to the psychological well-being of adolescents with NS. Studies suggest that a strong sense of MOL aids in coping with chronic illness stress and provides resilience against psychological distress (Marco et al., 2022). Additionally, recent research highlights that meaning in life is a significant positive predictor of adolescent self-control, a crucial factor in managing the demands of chronic conditions like NS (Liu et al., 2022). This relationship is further influenced by the level of perceived social support, which partially mediates the impact of MOL on self-control (Jönsson et al., 2020). This mediation underscores the importance of social support systems in helping adolescents with NS find significance in their experiences and manage their condition more effectively (Beanlands et al., 2017). Thus, examining MOL within the NS framework becomes crucial, not only for understanding how these adolescents find significance despite their condition’s challenges but also for identifying the supportive structures that best foster resilience and self-control.

Research on MOL in adolescents with chronic conditions, such as studies among Chinese adolescents, demonstrates the significant role of parent–child relationships and societal factors in shaping MOL. These studies reveal how family dynamics and societal acceptance contribute to adolescents’ sense of personal meaning, particularly in the face of chronic conditions like nephrotic syndrome (Shek et al., 2021). MOL pertains to the intrinsic sense of purpose and significance an individual derives from their experiences and relationships, which is crucial for psychological resilience and coping.

In contrast, Quality of Life (QOL) encompasses the broader spectrum of an individual’s overall well-being, including physical, psychological, and social domains. QOL research in adolescents with nephrotic syndrome underscores the multifaceted impact of the disease (Politano et al., 2020; Aronu et al., 2021; Menon et al., 2021; Abbasi et al., 2022). For example, Aronu et al. (2021) show a systematic review that establishes the reliability of generic QOL instruments in this demographic, revealing consistently low health-related quality of life (HRQoL) scores linked to prolonged disease duration and severe clinical phenotypes. The cross-sectional study by Menon et al. (2021) shows that while certain behavioral problems and severe complications can significantly affect QOL, regular follow-ups and supportive interventions can mitigate these effects. Additionally, the study Abbasi et al. (2022) on Iranian children with nephrotic syndrome finds that the condition affects physical, social, and educational fields of life, suggesting the need for comprehensive, multidisciplinary care. These studies collectively highlight the complex interplay of medical, psychological, and social factors affecting adolescents with nephrotic syndrome, emphasizing the need for holistic interventions that address both psychological well-being and overall quality of life.

Despite the broader context of QOL studies in various chronic conditions, research specifically addressing the Meaning of Life (MOL) in adolescents with nephrotic syndrome remains scarce. This gap highlights a critical need for focused investigation into how these adolescents find personal significance amidst their health challenges. Understanding MOL in the context of nephrotic syndrome, a condition that profoundly affects various life aspects (Huang et al., 2023), is crucial for developing targeted psychological interventions and support mechanisms tailored to this unique demographic (Ge et al., 2022).

Therefore, this study seeks to address this gap by exploring MOL in Chinese adolescents with NS through a comprehensive cross-sectional investigation and also understand the factors that affect MOL. By understanding how these patients perceive and find meaning in their lives amidst their health challenges, we aim to uncover insights that can guide more effective psychological interventions and support mechanisms tailored to this specific group, thereby contributing to their holistic care and treatment.

## 2 Methods

### 2.1 Study design and setting

The cross-sectional survey design was chosen for this study to efficiently capture the current psychological state and MOL levels of a specific cohort – adolescents with NS – at a single point in time. This approach was deemed most suitable due to its ability to provide a snapshot of the participants’ experiences and perceptions without the need for longitudinal follow-up. Additionally, the use of a paper-based questionnaire allowed for a standardized method of data collection across a large number of participants within a defined period.

The study focused on Chinese adolescent patients with NS in Baise City, Guangxi province, China, from January to December 2021. The Affiliated Hospital of Youjiang Medical University for Nationalities in Baise City was selected for patient recruitment through convenience sampling to include adolescents diagnosed with NS for investigating their level of MOL. The study adhered to the Declaration of Helsinki guidelines. Prior to commencement, informed consent was obtained from the participants’ guardians, and the study received approval from the hospital’s Ethics Committee (approval number: 2020110201).

The hospital was selected as the recruitment site due to its significant role in the healthcare system of Baise City. As a premier institution at the junction of Guangxi, Yunnan, and Guizhou provinces, it stands out for its high-level medical treatment, teaching, and research. Serving a diverse population, including a considerable number of adolescents, the hospital provides 4,000 beds and a wide range of medical services. This makes it an ideal location for accessing a representative adolescent sample, especially for a study focusing on a condition like NS. This hospital is renowned for its extensive expertise in the prevention and treatment of chronic kidney disease and has established itself as a significant national center for nephrology research. This reputation underscores its suitability as a prime site for recruiting a representative sample of adolescents with NS. Furthermore, the hospital’s commitment to research and its collaboration with academic institutions provide a supportive environment for conducting a study of this nature.

During data collection, researchers clearly explained the requirements for completing the questionnaires to patients and their families. To maintain the authenticity and integrity of the information, patients either filled out the questionnaires themselves or were assisted by family members or researchers, ensuring informed consent throughout the process. To optimize response rates, questionnaires were distributed during lunch breaks, and patients were asked to independently complete and return them. Returned questionnaires were anonymized and systematically managed. Those with missing values were excluded.

A “double check and double entry” method was employed by researchers to ensure data accuracy and reliability. This method involves two key steps: Firstly, ‘double check’ refers to having two independent researchers review the collected data. Each researcher checks the data for completeness, consistency, and accuracy, ensuring that any discrepancies or errors are identified and corrected. Secondly, ‘double entry’ involves entering the data into the database twice, typically by two different individuals. The two sets of entered data are then compared, and any inconsistencies are resolved. This method significantly reduces the likelihood of errors and increases the confidence in the accuracy of the data used for analysis.

### 2.2 Participants

One hundred and sixty adolescents with NS were recruited as participants from the NS patients who were admitted to the First Affiliated Hospital of Youjiang Medical University for Nationalities between January and December 2021. However, after applying the eligibility and exclusion criteria, the final number of participants was 150.

Eligibility Criteria: (1) Adolescents aged between 10 and 21 years at the time of the survey; (2) Participants must have been diagnosed with NS for a minimum duration of 1 year. This criterion ensures that the participants have adequate experience and familiarity with living with NS; (3) Participants should have a normal kidney function, indicated by a Glomerular filtration rate (GFR) of more than 90 mL/min/1.72m^2^. This criterion is crucial to focus on the impact of NS without the interference of kidney dysfunction; (4) Adolescents must possess the understanding and ability to answer the questions in the survey, ensuring meaningful engagement and valid responses. This was assessed through a preliminary screening process where participants completed a brief questionnaire designed to evaluate their comprehension and response abilities. Participants were asked to respond to several sample questions similar to those in the actual survey. Their responses were reviewed by a trained psychologist to ensure clarity, relevance, and coherence. Only those who demonstrated a clear understanding and ability to respond appropriately were included in the study. (5) Participants and their guardians (if minors) must express their willingness and cooperation to participate in the study.

The study included adolescents aged 10 to 21 years to capture a comprehensive view of the developmental spectrum within this group. This age range was chosen to align with the critical developmental stages relevant for the exploration of MOL in the context of chronic illness. Adolescents in this age bracket experience significant physical, psychological, and social changes, which can influence their perception of life meaning and quality of life. Including this broad age range allows us to understand how MOL and QOL vary across different stages of adolescence, from early adolescence (10–13 years) to late adolescence and young adulthood (19–21 years). To address potential variability within this cohort, we employed stratified analyses and included age as a covariate in our statistical models.

Exclusion Criteria: (1) Adolescents with NS secondary to other systemic diseases or conditions will be excluded; (2) Participants with significant comorbid physical, psychological, or obesity-related disorders will be excluded to isolate the effects of NS on MOL; (3) Adolescents with any active infection, edema, or those currently hospitalized at the time of enrollment will be excluded; (4) Adolescents with a low GFR or other indicators of impaired kidney function will be excluded; (5) Adolescents with obesity or any physical or psychological disability that could confound the study’s outcomes will be excluded.

These criteria aim to create a homogenous and representative sample of adolescents with NS, focusing on their experiences and perceptions related to MOL, while minimizing the influence of external variables.

### 2.3 Measures

#### 2.3.1 Demographic data

Participants provided detailed information on various sociodemographic characteristics, including age (recorded as continuous variables), gender (male or female), only-child status (whether the participant is an only child or not), family type (categorized as nuclear, extended, or single-parent), personal education level (classified as primary, middle school, or high school), and school status (categorized as in school, temporarily withdrawn, or permanently withdrawn). Additionally, left-behind status was identified, where left-behind children are those whose parents have migrated for work, typically to urban areas, leaving the children in the care of relatives (yes or no). Parents’ education level was recorded as the highest educational attainment of both parents, categorized as primary school, middle school, high school, or college and above. Annual family income was categorized into three brackets: less than 20,000 RMB, 20,000–60,000 RMB, and more than 60,000 RMB.

#### 2.3.2 Disease-related data

This encompassed the frequency of hospitalizations due to NS, duration of NS, and degree of edema. These medical aspects of NS are important to consider as they can have a direct impact on the psychological well-being of adolescents living with this condition.

#### 2.3.3 Purpose in life (PIL) scale

The PIL scale, developed by Crumbaugh and Maholick (1964) and translated by Song into Chinese version, was employed to assess the sense of MOL among adolescents with NS. The scale consists of 20 items that collectively evaluate vital aspects of life’s meaning and purpose, categorized into five dimensions: Passion for Life, Life Goals, Sense of Autonomy, Proactivity, and Future Aspirations. The scale is predominantly self-reported, with 18 out of the 20 items requiring personal reflection and responses from the participants.

Each item on the PIL scale is rated using a 7-point Likert scale, with 1 being the lowest score, indicating a lack of agreement or presence of the quality, and 7 as the highest score, signifying strong agreement or presence. The aggregate scores from these items are then interpreted into three distinct categories: scores below 92 indicate a significant lack of MOL, suggesting potential feelings of emptiness or lack of direction; scores ranging between 92 and 112 denote ambiguity in MOL, reflecting a state of exploration or seeking clarity; and scores exceeding 112 represent a clear and strong sense of purpose and MOL, typically associated with higher psychological resilience and well-being.

The reliability of the PIL scale used in this study is evidenced by a Cronbach’s alpha coefficient of 0.878. Cronbach’s α is a measure of reliability, or the degree to which a set of items measures a single unidimensional latent construct. A coefficient above 0.7 is generally considered acceptable, with higher values indicating greater reliability. Thus, the high value of Cronbach’s alpha coefficient signifies a strong internal consistency among the items, ensuring that the scale reliably measures the construct of MOL.

The PIL scale has been adapted and tested in various contexts to ensure its suitability for adolescents. In this study, special attention was given to ensure that the language and content of the scale were appropriate for younger participants. Prior to the main survey, a pilot test was conducted with a small group of adolescents aged 12 to 13 to confirm their comprehension of the items. Feedback from this pilot test indicated that the participants were able to understand and accurately respond to the questions.

#### 2.3.4 Psychological security (PS) scale

The PS scale, created by Cong and An (2004), plays a pivotal role in evaluating the psychological security of adolescents with NS. This scale features 16 items, intricately divided into two intertwined dimensions. The first dimension, Interpersonal Security, consists of eight items that assess an individual’s sense of safety and comfort in social interactions, capturing elements of trust, confidence, and ease in social environments. Given NS’s potential effects on social dynamics, understanding this aspect is vital for adolescents navigating their relationships. The second dimension, Certainty in control, also comprises eight items and focuses on the individual’s perceived control over and predictability in life, emphasizing feelings of command over one’s actions and life events. This dimension is particularly crucial for adolescents with NS, as it addresses the challenges posed by the unpredictability of their illness. Each item on the scale is rated on a 5-point Likert scale, enabling a comprehensive evaluation of psychological security, where higher scores indicate greater levels of security and stability in the psychosocial realm. The scale’s strong internal consistency, reflected in its Cronbach’s alpha coefficient of 0.873, underscores its reliability in effectively measuring the constructs of interpersonal security and certainty and control, providing valuable insights into the psychological well-being of adolescents with NS.

#### 2.3.5 Subjective well-being (SWB) scale

The SWB scale, developed by the U.S. National Center for Health Statistics and adapted into Chinese by scholar Duan Jianhua, is an extensive instrument designed to evaluate an individual’s happiness and overall subjective well-being (Yang et al., 2018). This scale encompasses 18 items, spread across six distinct dimensions: Concern for health, Vitality, Satisfaction and interest in life, Mood of melancholy or pleasure, Control over emotions and behavior, and Relaxation or tension. Each dimension plays a critical role in painting a comprehensive picture of an individual’s mental and emotional state, making it especially relevant for assessing adolescents with Nephrotic Syndrome, who may experience diverse emotional challenges due to their condition.

The scoring mechanism of the SWB scale is constructed to reflect the intensity or frequency of experiences and feelings noted in each item, with higher scores indicating a more positive state of subjective well-being. In the context of this study, the Chinese version of the SWB scale demonstrated excellent reliability, evidenced by a Cronbach’s α coefficient of 0.822. This coefficient, a measure of internal consistency, confirms the scale’s reliability in measuring subjective well-being consistently.

The inclusion of the SWB scale in our research provides a vital tool for understanding the varied impacts of Nephrotic Syndrome on the happiness and life satisfaction of adolescents. Its comprehensive nature and proven reliability are crucial for accurately assessing their mental and emotional health, thus informing the development of targeted interventions to enhance their overall well-being.

#### 2.3.6 Analysis of sociodemographic and disease-related covariates

In this study, a range of sociodemographic and disease-related factors were identified as covariates to understand if the observed effects were due to the primary variables, such as the impact of NS on psychological MOL, and not because of these other factors. Sociodemographic characteristics included age, gender, only-child status, family type, personal education level, school status, left-behind status, parents’ education level, and annual family income. Disease-related data included the frequency of hospitalizations due to NS, duration of NS, and degree of edema. By incorporating these covariates into the analysis, the study aimed to provide a comprehensive understanding of how various sociodemographic and disease-related factors interact with and influence the psychological aspects of adolescents with Nephrotic Syndrome.

### 2.4 Data analysis

Our study was retrospective, and the sample size was constrained by the available data; therefore, a post-hoc power calculation was performed by using PASS software. Based on our small-scale pilot study, we anticipated a 65% prevalence of low MOL among adolescents with NS. As this is pioneering research with no existing MOL data for adolescents with NS, we adopted a conservative approach, assuming a 50% prevalence of low MOL in the general adolescent population. This assumption provides the maximum variability in the data, which typically necessitates a larger sample size to detect a given effect. In turn, this approach tends to yield a more conservative estimate of the study’s power, ensuring robustness in our findings. Based on this assumption, we estimated a 96% power with α error of 0·05.

The initial phase of the analysis involved summarizing the collected data through descriptive statistics. This included computing the frequency, percentage, mean, standard deviation, and the range (minimum and maximum values) for each variable. The Shapiro–Wilk tests were also performed to assess if the data are normally distributed (Ghasemi and Zahediasl, 2012).

Multiple linear regression analysis was then utilized to explore the relationship between various independent variables and the MOL score derived from the PIL scale. This analysis accounted for potential confounding factors and identified predictors of MOL. Similar statistical methods, including linear regression and descriptive statistics, were applied to the scores from the PS scale. This allowed for the examination of factors influencing the sense of psychological security among participants. Also, the SWB scale data were analyzed using both descriptive and inferential statistical methods. The relationship between subjective well-being scores and other variables like demographic characteristics and disease-related factors was explored.

We employed Pearson correlation analysis for normally distributed data, and non-parametric Spearman correlation analysis for non-normally distributed data, to explore the relationships between scores of the PIL, PS, and SWB scales, as well as their respective dimensions. This approach was instrumental in uncovering how these varied psychological aspects are interrelated among adolescents diagnosed with NS. Multiple stepwise linear regression analysis was used to identify key factors influencing MOL, psychological security, and subjective well-being, thereby determining the most significant predictors for each psychological construct.

Chi-square tests for categorical variables and t-tests for continuous variables were conducted to assess the relationships between individual factors (like gender, family type) and scores from each of the scales. Data entry and management were performed using Excel software 2016. Statistical analysis was conducted using SPSS 26.0, with a significance level set at a *p*-value of less than 0.05.

## 3 Results

### 3.1 Demographic characteristics

As shown in Table 1, our study encompassed a cohort primarily composed of male participants (78.7%), spanning ages 12 to 21 years, with the average age of 17.8 ± 1.9 years and median age of 18. More than half of the participants’ age (55.3%) are between 18 and 21. A striking majority of these adolescents were not from single-child family (88.7%), suggesting the presence of siblings. Educational engagement was varied, with a sizable fraction actively attending school (45.3%) and a considerable portion not attending school (41.3%).

**Table 1:** Detailed demography and clinical characteristics of the adolescent patients with NS (*n* = 150).

Covariates	Classification	Frequency (percentage)	Covariates	Classification	Frequency (percentage)
Age (year)	<16	13 (8.7%)	Left-behind children	No	134 (89.3%)
	16–18	73 (48.6%)		Yes	16 (10.7%)
	>18	64 (42.7%)	Father’s education level	Primary school and below	41 (27.3%)
Gender	Male	118 (78.7%)		Junior high school	85 (56.7%)
	Female	32 (21.3%)		Vocational high school/senior high school and above	24 (16.0%)
Single--child family	No	133 (88.7%)	Maternal education level	Primary school and below	86 (57.3%)
	Yes	17 (11.3%)		Junior high school	41 (27.3%)
Family type	Single-parent family	16 (10.7%)		Vocational high school/senior high school and above	23 (15.4%)
	Nuclear family	78 (52.0%)	Annual family income (ten thousand yuan)	<2	82 (54.7%)
	Extended family	56 (37.3%)		2–6	36 (24.0%)
Personal education level	Junior high school and below	82 (54.6%)		>6	32 (21.3%)
	Vocational high school or senior high school	40 (26.7%)	Hospitalization frequency due to NS (times)	≤7	66 (44.0%)
	College degree or above	28 (18.7%)		>7	84 (56.0%)
School status	Persistence	68 (45.3%)	Duration of NS (months)	<24	67 (44.7%)
	Intermittent	13 (8.7%)		24–48	48 (32.0%)
	Medical leave of absence	7 (4.7%)		>48	35 (23.3%)
	Dropping out of school	62 (41.3%)	Severity of edema	Mild or no	77 (51.3%)
				Moderate and above	73 (48.7%)

The term ‘left-behind children’ refers to a unique demographic in China, denoting children who remain in rural regions while their parents migrate to urban areas for work. This study included a small percentage (10.7%) of such children, which may have implications for their social support systems and overall well-being.

When examining parental education, we observed a trend where a significant proportion of fathers (27.3%) had education levels of primary school and below, while this was even more pronounced among mothers (57.3%). Financially, the bulk of the families (54.7%) reported an annual low income of less than 20,000 yuan, highlighting economic challenges that may intersect with disease management.

Medical histories indicated a substantial burden of care, with over half of the participants (56.0%) being hospitalized more than seven times, and a significant duration of disease (23.3% over 48 months), pointing to the chronic nature of NS. Symptom severity varied, with almost an equal split between those with mild or no edema (51.3%) and those with moderate to severe edema (48.7%).

### 3.2 Assessment of MOL among adolescents with NS

**Figure 1:**
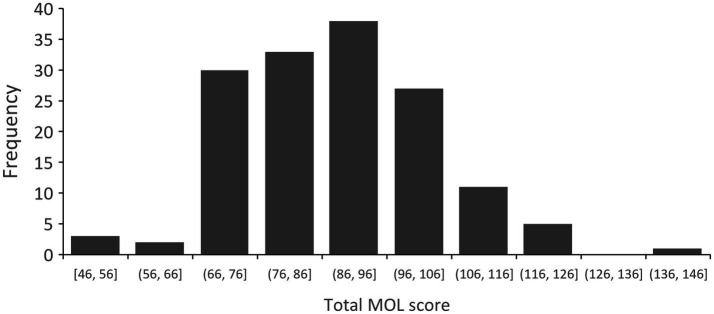
The distribution of total MOL score.

In this cohort of 150 patients, the MOL assessment revealed a general inclination toward a lower sense of MOL, as presented in Figure 1. The median MOL score for the cohort was 88, with an interquartile range of 77 to 99, notably below the threshold of 92, which statistically substantiates the prevalence of a significantly low level of MOL within the group (*t* = −2.735, *p* < 0.05). A small fraction, representing 11 cases (7.3%), scored above 112 points, signifying a high sense of MOL. The middle group, comprising 46 cases (30.7%), had scores ranging from 92 to 112 points. However, the majority, with 93 cases (62.0%), fell below the 92-point threshold, indicating a low sense of MOL. The distribution of MOL scores adhered to normality (*p* > 0.05) by conducting the Shapiro–Wilk test, suggesting no significant skewness or outliers affecting the overall data trend. This normality also validates the use of one-way ANOVA for further analysis.

Diving deeper into the individual dimensions from PIL scale that is used for assessing sense of MOL (Table 2), ‘Proactivity’ emerged as the dimension with the highest average score of MOL (5.01 ± 1.36), suggesting that despite overall lower MOL, participants exhibited a degree of passion or intense interest. Conversely, ‘Sense of Autonomy’ scored the lowest (3.53 ± 1.10), hinting at potential challenges in feeling independent or self-directed, which could reflect the chronic nature of NS and its impact on patients’ lives. The ‘Life Goals’ and ‘Future Aspirations’ dimensions showed moderately high MOL scores (4.73 ± 0.83 and 4.71 ± 1.29, respectively), indicating that aspirations and forward-looking perspectives were relatively maintained among the adolescents. ‘Passion for Life’ scored 4.22 ± 0.78, aligning fourth in the rank of dimensions.

**Table 2:** Scores of the five dimensions from PIL scale (*n* = 150,^−^*x* ± *s*).

Dimensions	Actual score range	Total point	Average	Ranking
Proactivity	2–14	10.0 ± 2.73	5.0 ± 1.36	1
Life goals	12–41	28.4 ± 5.00	4.7 ± 0.83	2
Future aspirations	4–14	9.4 ± 2.57	4.7 ± 1.29	3
Passion for life	20–55	33.7 ± 6.21	4.2 ± 0.78	4
Sense of autonomy	2–14	7.1 ± 2.19	3.5 ± 1.10	5

To explore the specific psychological impacts of NS on adolescents, discern potential relationships within the various PIL scale dimensions, and unveil factors influencing MOL to guide the creation of precise psychosocial interventions, a detailed correlation analysis of the distinct PIL scale dimensions was carried out. Prior to this analysis, the Shapiro–Wilk test was applied to assess the normality of the distribution of PIL scale dimensions. Except for ‘Passion for Life’ (*p* = 0.058), the dimensions deviated from normal distribution, with ‘Life Goals’, ‘Sense of Autonomy’, ‘Proactivity’, and ‘Future Aspirations’ yielding *p* values of 0.014, <0.001, <0.001, and < 0.001, respectively. Consequently, the non-parametric Spearman correlation was employed due to the non-normality of the data, and the findings are compiled in Table 3.

**Table 3:** Correlation analysis results of the five dimensions from PIL scale.

Dimensions	Proactivity	Life goals	Future aspirations	Passion for life	Sense of autonomy
Proactivity	1	0.668**	0.433**	0.669**	0.318**
Life goals	0.668**	1	0.463**	0.730**	0.261**
Future aspirations	0.433**	0.463**	1	0.591**	0.405**
Passion for life	0.669**	0.730**	0.591**	1	0.488**
Sense of autonomy	0.318**	0.261**	0.405**	0.488**	1

**p < 0.01.

The above correlation matrix reveals that ‘Proactivity’ is strongly linked with ‘Life Goals’ and ‘Passion for Life’, suggesting that an active stance toward life may be underpinned by clear goal-setting and a robust zest for living. Similarly, ‘Life Goals’ share a significant positive relationship with ‘Passion for Life’, highlighting that future-oriented objectives might enhance one’s enthusiasm for daily experiences. ‘Future Aspirations’ also exhibit a notable association with ‘Proactivity’ and ‘Passion for Life’, pointing to a synergy between looking ahead and engaging proactively with life’s opportunities. Interestingly, ‘Sense of Autonomy’—while correlating positively with all dimensions—shows a slightly lesser degree of association, indicating that while autonomy is an integral component of PIL, its influence is somewhat independent of the other dimensions. These interrelationships signal those interventions aimed at one domain, such as enhancing goal clarity, could potentially uplift other aspects of adolescents’ MOL, offering a multiplicative effect on their overall psychological well-being.

### 3.3 Influences on MOL scores: one-way ANOVA results

The ANOVA identified significant variations within specific demographic and clinical groups in terms of their MOL scores (*p* < 0.05), particularly concerning family type, school status, left-behind children status, annual family income, hospitalization frequency due to NS, and duration of NS (Table 4). Notably, single-parent families, disrupted school status, and being a left-behind child were associated with significantly lower MOL scores. For example, the average MOL scores for adolescents with disrupted school status and left-behind status are 83.7 and 73.8, much less than the threshold value of 92.

**Table 4:** Effect of general data on MOL in adolescent patients with NS (*n* = 150,*x¯* ± *s*).

Covariates	Classification	Frequency	Average MOL score	*t*/*F*	*P*-value
Age (year)	<16	13	95.8 ± 19.00	1.866	0.158
	16–18	73	88.8 ± 16.01		
	>18	64	86.9 ± 13.06		
Gender	Male	118	88.5 ± 15.80	−0.151	0.880
	Female	32	89.0 ± 12.95		
Single-child family	No	133	88.6 ± 14.84	−0.062	0.950
	Yes	17	88.8 ± 18.28		
Family type	Single-parent family	16	84.4 ± 19.60	7.705	**0.001**
	Nuclear family	78	85.1 ± 12.46		
	Extended family	56	94.6 ± 15.62		
Personal education level	Junior high school and below	82	86.1 ± 14.22	2.490	0.086
	Vocational high school or senior high school	40	91.3 ± 16.72		
	College degree or above	28	92.1 ± 14.89		
School status	Persistence	68	94.5 ± 16.01	7.559	**<0.001**
	Intermittent	13	83.7 ± 9.72		
	Medical leave of absence	7	90.4 ± 11.79		
	Dropping out of school	62	83.0 ± 13.20		
Left-behind children	No	134	90.4 ± 14.33	4.384	**<0.001**
	Yes	16	73.8 ± 14.46		
Father’s education level	Primary school and below	41	87.8 ± 13.90	0.495	0.611
	Junior high school	85	88.2 ± 16.24		
	Vocational high school/senior high school and above	24	91.4 ± 13.65		
Maternal education level	Primary school and below	86	87.8 ± 14.45	0.557	0.574
	Junior high school	41	88.7 ± 17.23		
	Vocational high school/senior high school and above	23	91.6 ± 14.33		
Annual family income (ten thousand yuan)	<2	82	85.6 ± 13.75	5.808	**0.004**
	2–6	36	88.7 ± 16.81		
	>6	32	96.1 ± 14.66		
Hospitalization frequency (times)	≤7	66	94.6 ± 15.95	4.589	**<0.001**
	>7	84	83.9 ± 12.79		
Duration of NS (months)	<24	67	93.5 ± 16.35	13.168	**<0.001**
	24–48	48	89.1 ± 11.06		
	>48	35	78.5 ± 13.03		
Severity of edema	Mild or no	77	87.8 ± 16.85	−0.705	0.482
	Moderate and above	73	89.5 ± 12.29		

The bold *p* values are those less than 0.05.

The study also has elucidated a series of influential elements. A clear age gradient was discernible, with the youngest cohort, those under 16, exhibiting the highest MOL scores, suggesting a greater resilience or perhaps a lesser comprehension of the disease’s implications. Conversely, as the adolescents matured, the MOL scores tended to wane, which might be attributed to the accruing social and academic pressures compounded by their chronic condition.

The family structure emerged as a pivotal determinant of MOL scores. Adolescents from single-parent families reported lower MOL scores compared to those from nuclear and extended family setups, underscoring the significance of familial support in managing chronic illness. Educational attainment, both adolescents and their parents, correlated positively with MOL scores, indicating that educational support and the nurturing environment it creates play a crucial role in fostering a positive outlook.

Socioeconomic status, as reflected by the annual family income, also had a bearing on the MOL scores, with higher income brackets correlating with higher scores. This linkage points to the stressors that financial strain can impose on families grappling with chronic disease management.

From a clinical perspective, the frequency of hospitalization and the overall duration of NS were inversely related to MOL scores. These findings indicate that the more intrusive and persistent the disease, the greater its toll on the adolescent’s sense of well-being. This underscores the imperative for a holistic approach to NS management that aligns clinical treatment with psychosocial support to ameliorate the disease’s impact over time.

Additionally, educational continuity emerged as a critical factor; consistent schooling was associated with higher MOL scores, highlighting the importance of minimizing educational disruptions for adolescents with NS. This suggests that interventions aimed at maintaining educational engagement during treatment could be beneficial in preserving the psychological well-being of these patients.

### 3.4 Multivariable regression analysis of MOL determinants

Following the initial observations from the one-way ANOVA, a detailed multivariable regression analysis was conducted to determine the specific impact of each variable on MOL scores among adolescents with NS. The analysis, presented in Table 5, shows the factors significantly influencing MOL scores.

**Table 5:** Regression analysis of sense of MOL in adolescent patients with NS.

Variable	Regression coefficient *(B)*	*SE*	*Beta*	*t*	*P*-value
Left-behind children	−9.828	3.520	−0.200	−2.792	**0.006**
Family type	6.022	1.630	0.254	3.695	**0.000**
School status	−2.341	0.763	−0.217	−3.067	**0.003**
Duration of NS	−2.066	0.731	−0.278	−2.826	**0.005**
Hospitalization frequency	−0.977	0.754	−0.128	−1.296	0.197
Annual family income	0.366	1.280	0.019	0.286	0.775

Adjusted *R*^2^ = 0.336, *F* = 13.551, *p* < 0.001. SE represents standard error. The Durbin-Watson statistic (1.52) indicates no major issues with autocorrelation. The bold *p* values are those less than 0.05.

The most significant determinant identified was the status of being a left-behind child, which showed a substantial negative impact on MOL scores (*B* = −9.828, *p* = 0.006). This finding highlights the profound psychological effect of familial separation and the absence of parental support on adolescents’ well-being. Family type emerges as a critical factor, with a positive regression coefficient for nuclear or extended family setups (*B* = 6.022, *p* < 0.001), reinforcing the importance of a supportive family environment in enhancing the MOL. Conversely, deviations from such family structures, including single-parent families, significantly reduce MOL scores (*B* = −2.341, *p* = 0.003), indicating the psychological challenges posed by these family dynamics.

School status, particularly disruptions in education due to NS, is negatively associated with MOL scores (*B* = −2.341, *p* = 0.003). This underscores the impact of disease-related educational interruptions on adolescents’ quality of life and points to the need for interventions to maintain educational continuity. The duration of NS is another significant predictor, with longer disease durations correlating with lower MOL scores (*B* = −2.066, *p* = 0.005), suggesting the cumulative adverse effect of prolonged disease management on adolescents’ psychological well-being. Interestingly, the frequency of hospitalization showed a negative but less significant impact on MOL scores (*B* = −0.977, *p* = 0.197), highlighting that while hospitalizations contribute to the disease burden, other factors have a more pronounced impact on adolescents’ MOL. Annual family income did not show a significant direct correlation with MOL scores (*B* = 0.366, *p* = 0.775), indicating that while financial resources are essential, the emotional and social support from family and educational stability plays a more critical role in influencing adolescents’ well-being in the context of NS.

This above analysis with an adjusted R2 of 0.336 and a significant *F*-value (*p* < 0.001) emphasizes the multifaceted nature of influences on adolescents’ MOL in the face of NS. It advocates for a holistic approach in the management of NS, one that encompasses not only clinical treatment but also robust support systems addressing the psychosocial, familial, and educational challenges encountered by these adolescents. This comprehensive approach aims to mitigate the disease’s impact, fostering an environment conducive to enhancing overall life quality and resilience in adolescents with NS.

### 3.5 Interrelationships of MOL and psychological dimensions

Understanding the intricate relationship between various psychological dimensions is crucial, as it can illuminate the broader impacts of life satisfaction and mental resilience on the overall well-being of patients with NS. Thus, the correlations between MOL score and other two psychological dimensions, i.e., SWB score and PS score are explored. Given the non-normal distribution of PS scores within our cohort (*p* < 0.001), the non-parametric Spearman correlation method was implemented.

The correlation analysis (Table 6) reveals a significant positive association between the MOL score and the SWB score (*r* = 0.76, *p* < 0.01), as well as between the MOL score and the PS score (*r* = 0.65, *p* < 0.01). Additionally, the SWB and PS scores are strongly correlated (*r* = 0.70, *p* < 0.01), indicating that higher MOL scores are associated with higher SWB and PS scores.

**Table 6:** Correlation analysis of MOL score with other two psychological assessments.

Outcomes	Total MOL score	Total SWB score	Total PS score
Total MOL score	1	0.76^**^	0.65^**^
Total SWB score	0.76^**^	1	0.70^**^
Total PS score	0.65^**^	0.70^**^	1

**P < 0.01.

In the assessment of SWB and PS among adolescents, the normality test indicated non-normal distributions for most dimensions, except Relaxation or tension (*p* = 0.232), necessitating the use of Spearman’s non-parametric correlation coefficients for analysis. A strong correlation (*r* = 0.634, *p* < 0.01) was observed between the two PS dimensions, indicating a significant relationship between stress factors in this demographic.

Table 7 presents the correlation analysis of the six dimensions of SWB scores. The ‘Concern for Health’ dimension, while fundamental to SWB, showed only weak to moderate correlations with the other dimensions, implying that health concerns may operate somewhat independently of other areas of subjective well-being. ‘Vitality’ emerged as a central component, showing strong correlations with ‘Mood of Melancholy or Pleasure’ (*r* = 0.699, *p* < 0.01) and moderate to strong correlations with other dimensions, indicating that a sense of energy and liveliness is closely intertwined with overall mood and emotional regulation.

**Table 7:** Correlation analysis of the six dimensions from SWB scale.

Dimensions	Concern for health	Vitality	Satisfaction and interest in life	Mood of melancholy or pleasure	Control over emotions and behavior	Relaxation or tension
Concern for health	1.000	0.118	0.135	0.290**	0.040	0.443**
Vitality	0.118	1.000	0.439**	0.699**	0.402**	0.524**
Satisfaction and interest in life	0.135	0.439**	1.000	0.427**	0.363**	0.315**
Mood of melancholy or pleasure	0.290**	0.699**	0.427**	1.000	0.477**	0.616**
Control over emotions and behavior	0.040	0.402**	0.363**	0.477**	1.000	0.336**
Relaxation or tension	0.443**	0.524**	0.315**	0.616**	0.336**	1.000

**P < 0.01.

‘Satisfaction and Interest in Life’ is moderately correlated with ‘Vitality’ and ‘Mood of Melancholy or Pleasure’ (*r* = 0.439 and *r* = 0.427, respectively, *p* < 0.01), suggesting that engagement and interest in life are significant contributors to the feeling of vitality and positive mood. The ‘Mood of Melancholy or Pleasure’ dimension has a strong correlation with ‘Relaxation or Tension’ (*r* = 0.616, *p* < 0.01), reinforcing the concept that mood is a significant factor influencing one’s ability to relax and feel pleasure.

The ‘Control over Emotions and Behavior’ dimension shows moderate correlations with ‘Vitality’ and ‘Mood of Melancholy or Pleasure’, indicating that the ability to manage emotions is linked with both a sense of vigor and a positive mood. Lastly, ‘Relaxation or Tension’ demonstrates strong correlations with ‘Mood of Melancholy or Pleasure’ and ‘Vitality’ (*r* = 0.616 and *r* = 0.524, respectively, *p* < 0.01), underscoring the impact of stress and relaxation on overall well-being and vitality.

These results highlight the interconnectedness of the SWB dimensions. While correlation does not imply causation, the findings suggest that improvements in one aspect of well-being could be associated with improvements in others. For example, higher vitality scores might coincide with better mood, while efforts to foster relaxation may have a positive impact on overall health concerns and emotional control. The significant correlations found within these dimensions underscore the complex and multi-faceted nature of subjective well-being in adolescents, particularly in the context of health-related experiences.

Within all the dimensions from PS scale and SWB scale, we found the top three dimensions that most correlate to the MOL score: certainty in control (*r* = 0.617, *p* < 0.01), Mood of melancholy or pleasure (*r* = 0.708, *p* < 0.01), and Vitality (*r* = 0.584, *p* < 0.01).

## 4 Discussion

In our study exploring MOL among 150 Chinese adolescents with NS, we unveiled several critical insights that directly align with our objective to pinpoint the factors that significantly influence MOL within this specific demographic. Notably, a substantial proportion of adolescents with NS (62.0%) recorded MOL scores below the established threshold, revealing a widespread inclination toward reduced life meaning in our cohort. Key demographic and social variables, such as the status of ‘left-behind children’, familial structure, educational disruptions, and the duration of NS, emerged as pivotal determinants of MOL, arranged by their degree of impact. Furthermore, our analysis highlighted profound correlations between MOL and other psychological dimensions, specifically SWB and PS, with a notably strong interrelation (*r* = 0.70, *p* < 0.01) between SWB and PS scores. Among these, ‘Certainty in Control’, ‘Mood of Melancholy or Pleasure’, and ‘Vitality’ were identified as the primary dimensions within the SWB and PS scales that exhibit the strongest correlation with MOL scores.

The finding that a significant fraction of adolescents with NS report lower MOL scores introduces a novel perspective into the existing body of research on chronic illnesses in young populations. This reduction in MOL scores among adolescents with NS can be attributed to several factors. First, the disease’s uncertainty and long-term management challenges erode patients’ confidence and patience, contributing to psychological distress, heightened anxiety, and depression, which ultimately affects their overall quality of life. Second, during adolescence—a critical period for self-image development—the adverse effects of glucocorticoids, such as moon face, buffalo hump, gynecomastia in males, and growth inhibition, create a pronounced difference between affected individuals and their healthy peers. This discrepancy can distort their perception of their social role, leading to feelings of isolation and a significant impact on their psychological well-being. Third, the prolonged treatment journey, accompanied by physical discomfort and financial strain, can lead adolescents to view themselves as burdens to their families. This perception may limit their focus to material aspects of life, hindering their engagement with higher spiritual and existential pursuits, and contributing to lower MOL scores.

The observation that adolescents with NS have lower sense of MOL aligns with the broader scholarly dialog on the psychological ramifications of chronic conditions on adolescents. Existing literature robustly illustrates how chronic diseases can profoundly undermine adolescents’ psychological health, ushering in heightened levels of anxiety, depression, and a diminished overall quality of life (Santos et al., 2016; Lacomba-Trejo et al., 2020; Koh et al., 2023). For example, the study by Lacomba-Trejo et al. (2020) delves into the determinants of anxiety and depression amongst adolescents facing chronic illnesses, shedding light on the substantial mental health challenges posed by these conditions. Similarly, research conducted by Santos et al. (2016) investigates the subjective experiences of adolescents living with chronic diseases and the associated psychosocial factors, offering valuable insights into the repercussions of chronic conditions on aspects such as school participation, engagement in leisure activities, and the general quality of life. These findings underline the substantial psychosocial burden chronic diseases impose on young individuals, thereby corroborating our study’s identification of reduced MOL scores in adolescents with NS as a significant area of concern.

Exploring the PIL scale’s individual dimensions to assess the sense of MOL, ‘Proactivity’ emerged as the highest scoring dimension. This finding underscores that, despite facing significant health obstacles, these adolescents exhibit a high degree of passion or an intense interest in engaging with life. Indeed, proactivity is strongly associated with motivation and positive outcomes in real-world settings. As a trait, it contributes significantly to positive life outcomes and satisfaction (Wolsink et al., 2019; Menon et al., 2021). The observed strong correlations between ‘Proactivity’, ‘Life Goals’, and ‘Passion for Life’ highlight the critical role of goal setting and an active stance toward life in bolstering their MOL. Conversely, the lower scores in ‘Sense of Autonomy’ shed light on the adolescents’ potential struggles with feeling independent, a reflection of the intricate challenges posed by managing a chronic condition like NS. These findings advocate for psychological interventions tailored to adolescents with NS to emphasize enhancing autonomy and proactivity. Recognizing that these individuals maintain a strong interest in life and clear aspirations, despite their health challenges, suggests that interventions aimed at nurturing their active engagement and supporting their life goals could significantly uplift their MOL. Such a focused approach would not only enrich their sense of meaning in life but also contribute meaningfully to their comprehensive care and treatment.

The association between demographic/social factors like ‘left-behind’ status, family structure, educational disruptions, and NS duration with MOL scores in adolescents underscores the complex link between chronic illness and their psychosocial health. This is in line with Pinquart and Shen’s findings (Pinquart and Shen, 2011) that chronically ill youths exhibit heightened depressive symptoms, which vary across conditions. The emphasis on stable family environments reflects the critical role of family support in managing chronic illness, resonating with several findings (Lum et al., 2017; Nielsen et al., 2017; Shek et al., 2021) such as from Lum et al. (2017) that chronic illness adversely impacts educational engagement, highlighting the importance of continuous education in these adolescents’ psychosocial well-being. The integrity of the family structure contributes positively to the warmth, care, and support received by patients, enhancing parent–child relations and family intimacy. Furthermore, our study’s findings on the adverse effects of NS severity and hospitalization frequency on MOL align with Modi et al. (2006) insights on the emotional toll of managing chronic conditions. Sawyer et al. (2007) also outline the challenges adolescents with chronic conditions face, including risky behaviors, stressing the need for interventions that address their comprehensive health needs. Overall, our research adds to the literature by detailing how specific factors affect MOL in adolescents with NS, advocating for integrated care approaches that include psychosocial support and educational assistance to improve their quality of life. It emphasizes the need for care strategies that consider these determinants to enhance the well-being of adolescents dealing with NS challenges.

Our analysis revealed significant correlations between MOL and other psychological constructs, notably SWB and PS, with a particularly robust association (*r* = 0.70, *p* < 0.01) between SWB and PS. Key dimensions driving this relationship included ‘Certainty in Control’, ‘Mood of Melancholy or Pleasure’, and ‘Vitality’. These findings emphasize the intricate connections between an individual’s perception of life meaning and their overall psychological health. Our findings resonate with research from Steinmayr et al. (2019) on the dynamic interplay of academic achievements, personality traits, and SWB in adolescents. They place on positive educational outcomes and personality traits enhancing SWB parallels our identification of ‘Certainty in Control’, ‘Mood of Melancholy or Pleasure’, and ‘Vitality’ as key influencers of MOL. This correlation underscores the integral role of fostering positive emotional states and a sense of control to enhance adolescents’ MOL and overall psychological well-being.

## 5 Implications for mental health

To enhance the life meaning and well-being of adolescents with NS, a comprehensive approach integrating psychosocial support into NS management is essential. Counseling and resilience workshops can mitigate feelings of isolation and improve self-esteem. Schools should offer flexible academic plans and online learning options to minimize educational disruptions, ensuring continued learning and engagement.

A family-centered care approach, offering resources and counseling to caregivers, can strengthen support systems and promote open communication about NS challenges. Special support for ‘left-behind’ adolescents through mentorship programs and healthcare access is crucial due to their unique challenges. Empowering adolescents with NS to participate in their care through goal setting and self-management enhances their sense of autonomy and engagement.

Training healthcare providers and educators on the psychosocial impacts of NS ensures they are equipped to support these adolescents effectively. Implementing these strategies through a collaborative approach involving healthcare, education, and family support can significantly improve the quality of life and psychological well-being of adolescents with NS.

## 6 Limitations of the study

The study presented here, while offering valuable insights into the psychological well-being and MOL among adolescents with NS, is subject to several limitations that underscore areas for future research. Conducted in a single center, the study’s findings may not be entirely representative of the broader population of adolescents with NS. Recruiting participants from multiple centers could enhance the generalizability of the results, offering a more comprehensive understanding of the psychological impacts of NS across different demographic and geographic contexts.

A notable limitation of the current research is its predominant male participant cohort, comprising 78.7% of the study sample. This gender disparity raises questions about the applicability of the findings to female adolescents with NS, who may experience the condition and its psychological implications differently. Future studies should aim to achieve a more balanced gender representation to explore potential differences in MOL and psychological well-being between male and female adolescents with NS.

The low family income level of participants in this study is another factor that warrants consideration. Given the potential influence of socioeconomic status on access to healthcare, treatment adherence, and overall quality of life, future research should include participants from a broader range of income levels. This would allow for a more nuanced analysis of how socioeconomic factors impact the psychological outcomes of adolescents with NS.

Additionally, the cross-sectional nature of this study limits its ability to establish cause-effect relationships. Longitudinal or prospective cohort studies could provide deeper insights into the dynamic interplay between NS and psychological well-being over time, offering a clearer understanding of causality and the long-term effects of living with NS.

The use of a limited set of psychological assessment scales in this study also points to an area for expansion in future research. Employing a wider array of scales to evaluate different aspects of psychological health could yield a more comprehensive picture of the psychological landscape of adolescents with NS. Comparative analyses using diverse psychological instruments would enrich the understanding of the multifaceted nature of psychological experiences among this population.

### 6.1 Summary

Overall, this study illuminates the psychological dimensions of living with NS in adolescence but also underscores the need for further research. It is essential to expand the scope by including a more diverse and representative sample, adopting longitudinal study designs, and using a broader range of psychological assessment tools. These steps will deepen our understanding of the impact of NS on adolescents’ lives. Future research directions will refine our knowledge and lead to more targeted and effective interventions to enhance the psychological well-being of adolescents with NS.

## 7 Conclusion

The study on adolescents with NS has uncovered critical insights into their psychological well-being, particularly regarding their sense of MOL. Key findings reveal that a significant proportion of these adolescents experience a diminished MOL, impacted by factors such as familial structures, educational disruptions, and the chronicity of NS. Notably, ‘left-behind’ status, disrupted schooling, and extended disease duration emerge as significant determinants, underscoring the importance of social and environmental contexts on adolescents’ psychological health.

Further, strong correlations between MOL and other psychological dimensions like SWB and PS highlight the interconnectedness of these constructs. Key psychological aspects identified, including ‘Certainty in Control’, ‘Mood of Melancholy or Pleasure’, and ‘Vitality’, suggest targeted areas for intervention.

However, it is important to note that the conclusion regarding the younger population, specifically those under 16 years old, should be treated with caution due to the small sample size (only 13 participants out of 150). This limitation suggests that the findings related to this subgroup may not be as robust and should be interpreted carefully.

In conclusion, this study emphasizes the necessity for a holistic care approach that integrates psychosocial support, addressing both medical and psychological needs of adolescents with NS. Such comprehensive care strategies, involving collaborative efforts among healthcare providers, educators, and families, are crucial for improving the overall well-being and quality of life for adolescents facing the challenges of NS.

## Data availability statement

The original contributions presented in the study are included in the article/supplementary material, further inquiries can be directed to the corresponding authors.

## Ethics statement

The studies involving humans were approved by the Ethics Committee of the Affiliated Hospital of Youjiang Medical University for Nationalities. The studies were conducted in accordance with the local legislation and institutional requirements. Written informed consent for participation in this study was provided by the participants’ legal guardians/next of kin.

## Author contributions

YL: Conceptualization, Methodology, Project administration, Writing – original draft. RH: Data curation, Visualization, Writing – review & editing. XL: Data curation, Investigation, Resources, Writing – review & editing. SM: Formal analysis, Software, Validation, Writing – review & editing. ZH: Data curation, Resources, Writing – review & editing. JT: Investigation, Resources, Writing – review & editing. LY: Software, Validation, Writing – review & editing. YX: Formal analysis, Investigation, Writing – review & editing. XL: Funding acquisition, Project administration, Supervision, Writing – review & editing.
